# Comment on ‘Adherence to Physical Activity and Incident Mobility Disability in Older Adults With Mobility Limitations’ by Alvarez‐Bustos et al.

**DOI:** 10.1002/jcsm.70071

**Published:** 2025-09-24

**Authors:** Yuxia Yang, Meijuan Yuan, Qing Kong, Zhiwei Peng, Yucheng Zhang

**Affiliations:** ^1^ School of Medicine of Yangzhou University Yangzhou China; ^2^ Northern Jiangsu People's Hospital, Affiliated to Yangzhou University Yangzhou China; ^3^ Department of Orthopaedics The Second Affiliated Hospital of Xuzhou Medical University Xuzhou China; ^4^ Department of Orthopaedics Changshu Hospital Affiliated to Nanjing University of Chinese Medicine Suzhou China; ^5^ Yangzhou University Yangzhou China

**Keywords:** frailty, implementation science, mobility disability, physical activity adherence, reimbursement models

Dear Editor,

While the clinical implications are clear, translating these findings into public health and community‐based interventions warrants further exploration. Here, we discuss three actionable dimensions to amplify the societal impact of this research.

First, Scalable Adherence Monitoring via Digital Health Technologies [1]: The study's categorization of adherence (BR, MR, AR) highlights the critical need for real‐time adherence tracking. Wearable sensors (e.g., ActiGraph, Fitbit) and AI‐driven platforms can objectively monitor PA frequency, intensity and duration in community settings. For instance, remote coaching apps with automated feedback loops, similar to the SPRINTT trial's technological support, could personalize adherence goals based on baseline SPPB scores. Integrating such tools into existing community health programmes (e.g., the WHO's Integrated Care for Older People, ICOPE) would democratize access, particularly in rural or resource‐limited areas [1].

Second, Community Health Workers as Adherence Facilitators [2]: Participants with SPPB 3–7 benefitted most from > 3 sessions/week, yet sustaining high adherence remains challenging. Task‐shifting to community health workers (CHWs) could bridge this gap. CHWs trained in geriatric exercise protocols can conduct home visits, deliver nutritional counselling and foster social accountability through peer‐support groups. Embedding CHWs within primary care networks would operationalize the study's adherence framework while addressing socioeconomic barriers [2].

Third, Policy Integration Regarding Reimbursement and Infrastructure: The differential benefit of AR adherence (HR: 0.33 for SPPB 3–7) underscores the need for policy‐level changes. Medicare's current reimbursement for ‘exercise as medicine’ is limited to cardiac rehabilitation. Expanding coverage to include multimodal PA programmes for PF&S—with incentives tied to adherence metrics—would incentivize healthcare systems to adopt SPRINTT‐like interventions [3]. Concurrently, investing in public infrastructure (e.g., safe walking paths, subsidized community gyms) could reduce environmental barriers to adherence, aligning with the WHO's ‘Decade of Healthy Ageing’ goals.

In conclusion, Alvarez‐Bustos et al.'s work provides a robust template for combating mobility disability. By leveraging digital tools, community networks and policy reform, we can transform adherence from a clinical variable into a public health priority.

## Conflicts of Interest

The authors declare no conflicts of interest.
